# Supplementary material for: Three-dimensional mapping of the altermagnetic spin splitting in CrSb
Source: Nat Commun. 2025 Feb 7;16:1442. doi: 10.1038/s41467-025-56647-7 (PMC11805911; doi:10.1038/s41467-025-56647-7)
Supplement: Supplementary file 1 — Supplementary Information [file 41467_2025_56647_MOESM1_ESM.pdf]

Supplementary information for:

Three-dimensional mapping of the altermagnetic spin splitting in CrSb

Guowei Yang[*1], Zhanghuan Li[†2], Sai Yang[3], Jiyuan Li[3], Hao Zheng[1], Weifan Zhu[1], Ze Pan[1], Yifu Xu[1], Saizheng Cao[1], Wenxuan Zhao[4], Anupam Jana[5,6], Jiawen Zhang[1], Mao Ye[7], Yu Song[1], Lun-Hui Hu[1], Lexian Yang[4], Jun Fujii[5], Ivana Vobornik[5], Ming Shi[1], Huiqiu Yuan[1,8], Yongjun Zhang[‡3], Yuanfeng Xu[§1] and Yang Liu[¶1,8]

[1]*Center for Correlated Matter and School of Physics, Zhejiang University, Hangzhou 310058, China*
[2]*Beijing National Laboratory for Condensed Matter Physics, Institute of Physics, Chinese Academy of Sciences, Beijing 100190, China*
[3]*Hubei Key Laboratory of Photoelectric Materials and Devices, School of Materials Science and Engineering, Hubei Normal University, Huangshi 435002, China*
[4]*State Key Laboratory of Low Dimensional Quantum Physics, Department of Physics, Tsinghua University, Beijing 100084, China*
[5]*CNR-IOM, TASC Laboratory, Area Science Park-Basovizza, Trieste 34139, Italy*
[6]*International Center for Theoretical Physics (ICTP), Trieste 34151, Italy*
[7]*Shanghai Synchrotron Radiation Facility, Shanghai Advanced Research Institute, Chinese Academy of Sciences, Shanghai 201204, China*
[8]*Collaborative Innovation Center of Advanced Microstructures, Nanjing University, Nanjing 210093, China*

This supplementary document contains the following parts:
I. Details of tight-binding model analysis, which includes Section 1-3, Table 1-4 and Figs. S1-S4, S13.
II. Additional sample characterization, which includes Fig. S5.
III. Additional DFT calculations, which include Figs. S6, S7, S9, S11, S12.
IV. Additional ARPES data and comparison with calculations, which include Figs. S8, S10.

1 Tight-binding Model

The magnetic crystal structure of CrSb is characterized by the magnetic space group $P6_3'/m'm'c$. As discussed in the main text, the low-energy band structure of CrSb in its magnetic phase is mainly contributed by the d orbitals on Cr, which have weak spin-orbit coupling (SOC). Therefore, we can ignore the SOC effect. In the following, we build a tight-binding Hamiltonian using its corresponding spin group symmetry to analyze the spin-splitting magnitude in the altermagnet CrSb. For each spin component in CrSb, its Hamiltonian is characterized by the spinless space group $P\bar{3}m1$ (#164), containing three generators C_{3z}, inversion P, and C_{2x}. Then, the two Hamiltonians with different spins are related by a composed symmetry $[C_2^s \| \{M_z|(0,0,\frac{1}{2})\}]$, where C_2^s is a spin-flip operation and $\{M_z|(0,0,\frac{1}{2})\}$ is a mirror reflection.

[*]These authors contributed equally to this paper
[†]These authors contributed equally to this paper
[‡]yjzhang@hbnu.edu.cn
[§]y.xu@zju.edu.cn
[¶]yangliuphys@zju.edu.cn

Table 1: Occupied Wyckoff positions in the crystal structure of CrSb. For each spin sublattice, it crystallizes in the space group $P\bar{3}m1$.

Atom	Wyckoff position	Coordinates	Site symmetry
Cr_1	1a	(0,0,0)	D_{3d}
Cr_2	1b	$(0,0,\frac{1}{2})$	D_{3d}
Sb	2d	$(\frac{1}{3},\frac{2}{3},z),(\frac{2}{3},\frac{1}{3},-z)$	C_{3v}

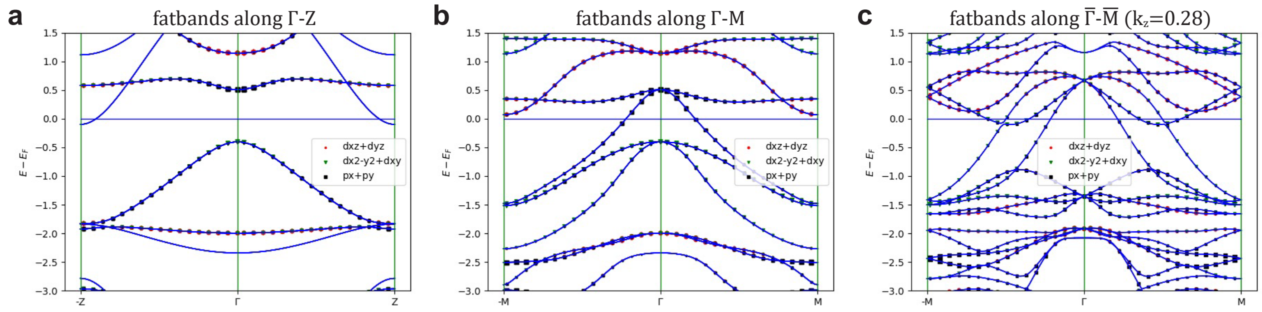

Fig. S1: The orbital-resolved magnetic band structures along **a** $\Gamma - Z(k_1 = 0$ and $k_2 = 0)$, **b** $\Gamma - M$ $(k_z = 0)$, and **c** $\bar{\Gamma} - \bar{M}$ $(k_z = 0.28)$. Here k_1, k_2 and k_z (or k_3) are along the direction of reciprocal lattice vectors. All the band structures are calculated without SOC. The weights of d_{xz} and d_{yz} orbitals on Cr, $d_{x^2-y^2}$ and d_{xy} orbitals on Cr, and p_x and p_y orbitals on Sb are plotted with red circles, green triangles, and black squares, respectively.

In this section, we build an eight-band tight-binding (TB) model for CrSb and analyze the origin of the large spin splitting along the $\Gamma - M$ direction. As plotted in Fig. S1, from ab initio calculations, we find that the band structures near the Fermi level (E_F) are dominated by the $d_{xz}, d_{yz}, d_{x^2-y^2}$, and d_{xy} orbitals on Cr atoms. In Table 1, we tabulate the occupied Wyckoff positions (in space group $P\bar{3}m1$) for the spin-up Hamiltonian of CrSb, where the site symmetry of Cr is D_{3d}. For point group D_{3d}, both the orbitals (d_{xz}, d_{yz}) and $(d_{xy}, d_{x^2-y^2})$ have the same symmetry properties, corresponding to a two-fold irreducible presentation (irrep) E_g. Without loss of generality, we use the two orbitals ϕ_1 and ϕ_2 as the basis of irrep E_g to construct the tight-binding Hamiltonian. Note that the representation matrices of $(d_{xy}, d_{x^2-y^2})$ are the same with those of (d_{xz}, d_{yz}) under point group D_{3d}. Specifically, the creation operators of the spin-up atomic orbitals read

$$\hat{c}^\dagger_{\mathbf{R},Cr_1,\phi_1,\uparrow}, \hat{c}^\dagger_{\mathbf{R},Cr_1,\phi_2,\uparrow}, \hat{c}^\dagger_{\mathbf{R},Cr_2,\phi_1,\uparrow}, \hat{c}^\dagger_{\mathbf{R},Cr_2,\phi_2,\uparrow}, \tag{1}$$

where $\mathbf{R}$ is the lattice vector. Here Cr_1 and Cr_2 denotes two Cr atoms with different Wyckoff positions (see Table 1). The four spin-down creation operators can be obtained by the spin group symmetry $\hat{P} = [C_2^s \| \{M_z | (0,0,\frac{1}{2})\}]$,

$$\begin{aligned} \hat{P}\hat{c}^\dagger_{\mathbf{R},Cr_1,\phi_1,\uparrow} &= -\hat{c}^\dagger_{\mathbf{R},Cr_2,\phi_1,\downarrow} \\ \hat{P}\hat{c}^\dagger_{\mathbf{R},Cr_1,\phi_2,\uparrow} &= -\hat{c}^\dagger_{\mathbf{R},Cr_2,\phi_2,\downarrow} \\ \hat{P}\hat{c}^\dagger_{\mathbf{R},Cr_2,\phi_1,\uparrow} &= -\hat{c}^\dagger_{\mathbf{R},Cr_1,\phi_1,\downarrow} \\ \hat{P}\hat{c}^\dagger_{\mathbf{R},Cr_2,\phi_2,\uparrow} &= -\hat{c}^\dagger_{\mathbf{R},Cr_1,\phi_2,\downarrow} \end{aligned} \tag{2}$$

Then, the 8-band Hamiltonian is

$$H = \sum_{\mathbf{R},\mathbf{R}',\tau,\tau',\alpha,\beta,s} \hat{c}^\dagger_{\mathbf{R}+\tau,\alpha,s} t^{\alpha s,\beta s}_{\mathbf{R}+\tau,\mathbf{R}'+\tau'} \hat{c}_{\mathbf{R}'+\tau',\beta,s}, \tag{3}$$

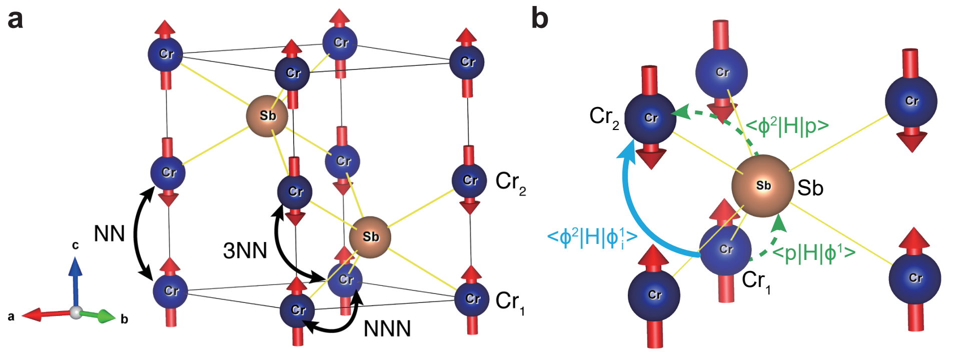

Fig. S2: **a** Chemical bonds between Cr and Sb atoms. The NN, NNN, and 3NN Cr-Cr bonds are indicated by the black arrow lines. **b** The dominant assistant hopping process between the 3NN Cr atoms at $(0,0,0)$ and $(1,1,\frac{1}{2})$, which is mediated by the Sb at $(\frac{1}{3},\frac{2}{3},\frac{1}{4})$.

where τ (τ') is the atom index representing Cr_1 or Cr_2, $\alpha(\beta)$ is the orbital index, and s is the spin index.

As we have ignored the spin-orbit coupling in Eq. 3, the Hamiltonian in reciprocal space takes the following form,

$$H(\mathbf{k}) = \begin{bmatrix} H^{\uparrow}(\mathbf{k}) & 0_{4\times4} \\ 0_{4\times4} & H^{\downarrow}(\mathbf{k}) \end{bmatrix} = \sum_{i=0,3}\sum_{j,m=0,1,2,3} f_{ijm}(\mathbf{k})\Gamma_{ijm} \tag{4}$$

where $\Gamma_{ijm} = s_i \otimes \sigma_j \otimes \tau_m \equiv s_i\sigma_j\tau_m$. The Pauli matrices $s_{i=0,1,2,3}$ (or σ_i or τ_i) represent the space of spin (or the two Cr sites or the two ϕ orbitals).

Let us consider the nearest neighbor (NN), the next nearest neighbor (NNN), and the third nearest neighbor (3NN) kinetic terms. As shown in Fig. S2a, the NN and 3NN bonds connect the two sublattices of Cr_1 and Cr_2, and the NNN bonds connect the Cr atoms within the same sublattice. There are two NN bonds for each Cr site, corresponding to $\mathbf{d} = \mathbf{R} + \tau - \mathbf{R}' - \tau' = (0,0,\pm\frac{1}{2})$. Each Cr site has six NNN bonds, corresponding to the lattice vectors $\mathbf{d} = (1,1,0),(1,0,0),(0,1,0),(0,-1,0),(-1,0,0),(-1,-1,0)$. For each Cr site, there are 12 3NN bonds, corresponding to $\mathbf{d} = (1,1,\frac{1}{2})$, $(1,1,-\frac{1}{2})$, $(1,0,\frac{1}{2})$,$(1,0,-\frac{1}{2})$, $(0,1,\frac{1}{2})$, $(0,1,-\frac{1}{2})$, $(0,-1,\frac{1}{2})$, $(0,-1,-\frac{1}{2})$, $(-1,0,\frac{1}{2})$, $(-1,0,-\frac{1}{2})$, $(-1,-1,\frac{1}{2})$, $(-1,-1,-\frac{1}{2})$. Under this approximation, we perform Fourier transformation on the Hamiltonian in Eq. 3, and derive the Hamiltonian along $\Gamma - M$ direction as follows:

$$\begin{aligned} H(\mathbf{k}) &= H_{on-site} + H_{NN} + H_{NNN} + H_{3NN} \\ &= H(\mathbf{r}) + \sum_{\mathbf{R}\in NN} e^{i\mathbf{k}\cdot\mathbf{R}} H(\mathbf{r\text{-}R}) + \sum_{\mathbf{R}\in NNN} e^{i\mathbf{k}\cdot\mathbf{R}} H(\mathbf{r\text{-}R}) + \sum_{\mathbf{R}\in 3NN} e^{i\mathbf{k}\cdot\mathbf{R}} H(\mathbf{r\text{-}R}) \\ &= f_{000}(\mathbf{k})\Gamma_{000} + f_{001}(\mathbf{k})\Gamma_{001} + f_{003}(\mathbf{k})\Gamma_{003} + f_{010}(\mathbf{k})\Gamma_{010} + \\ &\quad f_{011}(\mathbf{k})\Gamma_{011} + f_{013}(\mathbf{k})\Gamma_{013} + f_{330}(\mathbf{k})\Gamma_{330} + f_{331}(\mathbf{k})\Gamma_{331} + \\ &\quad f_{333}(\mathbf{k})\Gamma_{333} + f_{311}(\mathbf{k})\Gamma_{311} + f_{313}(\mathbf{k})\Gamma_{313} \end{aligned} \tag{5}$$

Here, we derive as follows the coefficients $f_{ijk}(\mathbf{k})$ along the $\Gamma - M$ ($\mathbf{k} = (k_1, 0, k_3)$) direction,

$$
\begin{aligned}
f_{000}(\mathbf{k}) &= \frac{1}{2}(e_1 + e_2) - \frac{1}{3}\left(\sqrt{3}r_1 - 3r_2 - \sqrt{3}r_3 - 3r_4\right)[2\cos(k_1) + 1] \\
f_{001}(\mathbf{k}) &= (r_1 - r_3)[\cos(k_1) - 1] \\
f_{003}(\mathbf{k}) &= \frac{(r_1 - r_3)[\cos(k_1) - 1]}{\sqrt{3}} \\
f_{010}(\mathbf{k}) &= 2[2(q_1 + q_4)\cos(k_1) + q_1 + q_4 + t_1]\cos\left(\frac{k_3}{2}\right) \\
f_{011}(\mathbf{k}) &= 2\sqrt{3}(q_4 - q_1)\sin^2\left(\frac{k_1}{2}\right)\cos\left(\frac{k_3}{2}\right) \\
f_{013}(\mathbf{k}) &= 2(q_4 - q_1)\sin^2\left(\frac{k_1}{2}\right)\cos\left(\frac{k_3}{2}\right) \\
f_{330}(\mathbf{k}) &= \frac{1}{2}(e_1 - e_2) - \frac{1}{3}\left(\sqrt{3}r_1 - 3r_2 + \sqrt{3}r_3 + 3r_4\right)[2\cos(k_1) + 1] \\
f_{331}(\mathbf{k}) &= (r_1 + r_3)[\cos(k_1) - 1] \\
f_{333}(\mathbf{k}) &= \frac{(r_1 + r_3)[\cos(k_1) - 1]}{\sqrt{3}} \\
f_{311}(\mathbf{k}) &= 3(q_2 + q_3)\sin(k_1)\sin\left(\frac{k_3}{2}\right) \\
f_{313}(\mathbf{k}) &= \sqrt{3}(q_2 + q_3)\sin(k_1)\sin\left(\frac{k_3}{2}\right)
\end{aligned} \tag{6}
$$

The free parameters in Eq. 6 are defined as follows:

- $e_i (i = 1, 2)$ is the onsite energy of the orbitals ϕ_1 and ϕ_2 on Cr_i.
- t_1 is the kinetic hopping strength between two ϕ_1 (or the equivalent ϕ_2) orbitals on two NN Cr atoms.
- $r_i (i = 1, 2, 3, 4)$ is the kinetic hopping between two NNN Cr atoms, whose respective coordinates are $R_1 = (0, 0, 0)$ and $R_2 = (1, 1, 0)$. Specifically, $r_1 = \langle\phi_1, Cr_1, R_1|\hat{H}|\phi_2, Cr_1, R_2\rangle$, $r_2 = \langle\phi_1, Cr_1, R_1|\hat{H}|\phi_1, Cr_1, R_2\rangle$, $r_3 = \langle\phi_2, Cr_2, R_1|\hat{H}|\phi_1, Cr_2, R_2\rangle$, $r_4 = \langle\phi_2, Cr_2, R_1|\hat{H}|\phi_2, Cr_2, R_2\rangle$.
- q_i(i=1,2,3,4) is the 3NN kinetic hopping from $R_1 = (0, 0, 0)$ to $R_2 = (1, 1, \frac{1}{2})$, defined as $q_1 = \langle\phi_1, Cr_1, R_1|\hat{H}|\phi_1, Cr_2, R_2\rangle$, $q_2 = \langle\phi_1, Cr_1, R_1|\hat{H}|\phi_2, Cr_2, R_2\rangle$, $q_3 = \langle\phi_2, Cr_1, R_1|\hat{H}|\phi_1, Cr_2, R_2\rangle$, $q_4 = \langle\phi_2, Cr_1, R_1|\hat{H}|\phi_2, Cr_2, R_2\rangle$.

To have an altermagnetic spin splitting (and band splitting), both the space-time reversal symmetry $[T\|P]$ and the translational spin-rotation symmetry $[C_2^s\|\vec{t}]$ ($\vec{t}$ is a fractional lattice vector) are necessarily broken in the analytic Hamiltonian in Eq. 5. As mentioned in the main text, either of the above two symmetries guarantees a spin-degenerate band structure. In Eqs. 7-8, $[T\|P]$ and $[C_2^s\|\vec{t}]$ are defined using the orbital basis in Eqs. 1-2. We find that the first nine terms in Eq. 5 commute with the symmetry $[C_2^s\|\vec{t}]$ and they do not give rise to the spin splitting along $\Gamma - M$ direction. In contrast, the last two terms, i.e. $f_{311}(\mathbf{k})\Gamma_{311}$ and $f_{313}(\mathbf{k})\Gamma_{313}$ which are proportional to $q_2 + q_3$, break both of the two spin group symmetries. Therefore the Hamiltonian in Eq. 5 has a finite spin splitting along $\Gamma - M$ direction when the 3NN hopping strength $q_2 + q_3$ is non-zero.

$$[T\|P] = \begin{bmatrix} 0 & 0 & 0 & 0 & 1 & 0 & 0 & 0 \\ 0 & 0 & 0 & 0 & 0 & 1 & 0 & 0 \\ 0 & 0 & 0 & 0 & 0 & 0 & 1 & 0 \\ 0 & 0 & 0 & 0 & 0 & 0 & 0 & 1 \\ 1 & 0 & 0 & 0 & 0 & 0 & 0 & 0 \\ 0 & 1 & 0 & 0 & 0 & 0 & 0 & 0 \\ 0 & 0 & 1 & 0 & 0 & 0 & 0 & 0 \\ 0 & 0 & 0 & 1 & 0 & 0 & 0 & 0 \end{bmatrix} K = s_1\sigma_0\tau_0 K, \text{ where } K \text{ is complex conjugate operator.} \tag{7}$$

$$[C_2^s\|\vec{t} = (0,0,1/2)] = \begin{bmatrix} 0 & 0 & 0 & 0 & 0 & 0 & 1 & 0 \\ 0 & 0 & 0 & 0 & 0 & 0 & 0 & 1 \\ 0 & 0 & 0 & 0 & 1 & 0 & 0 & 0 \\ 0 & 0 & 0 & 0 & 0 & 1 & 0 & 0 \\ 0 & 0 & 1 & 0 & 0 & 0 & 0 & 0 \\ 0 & 0 & 0 & 1 & 0 & 0 & 0 & 0 \\ 1 & 0 & 0 & 0 & 0 & 0 & 0 & 0 \\ 0 & 1 & 0 & 0 & 0 & 0 & 0 & 0 \end{bmatrix} = s_1\sigma_1\tau_0 \tag{8}$$

By fitting the band structure from ab initio calculations, especially the dispersion along $\Gamma - M$ direction near the Fermi level, we obtain the parameters in Eq. 6, as tabulated in Table 2. The comparison between band structures from ab initio calculation and the tight-binding model is plotted in Fig. S3.

Table 2: Parameters in the TB model (Eq. 6) obtained by fitting to the ab initio calculation. The unit is eV.

e_1	e_2	t_1	r_1	r_2	r_3	r_4	q_1	$q_2 + q_3$	q_4
-3.5883	-1.4522	1.6	-0.1	0.305	0.1	-0.12	-0.0184	0.195	0.1942

2 Derivation of the 3NN hopping parameters

In the above section, we have analyzed qualitatively the origin of the spin/band splitting along $\Gamma - M$ direction, which is proportional to the kinetic hopping between the two d orbitals on the two 3NN Cr atoms. Usually, the Wannier functions of d orbitals are very local, and the hopping between them is tiny. While in Table 2, the fitted 3NN hopping is in the same order as the NNN hopping, which is counterintuitive. To have a deep understanding of this, it is necessary to derive the 3NN hopping parameters from ab initio calculations.

Based on the Wannier calculations, we find that the bands near E_F come from a strong hybridization between the two E_g orbitals (d_{xz}, d_{yz}) and $(d_{xy}, d_{x^2-y^2})$, and they can be characterized

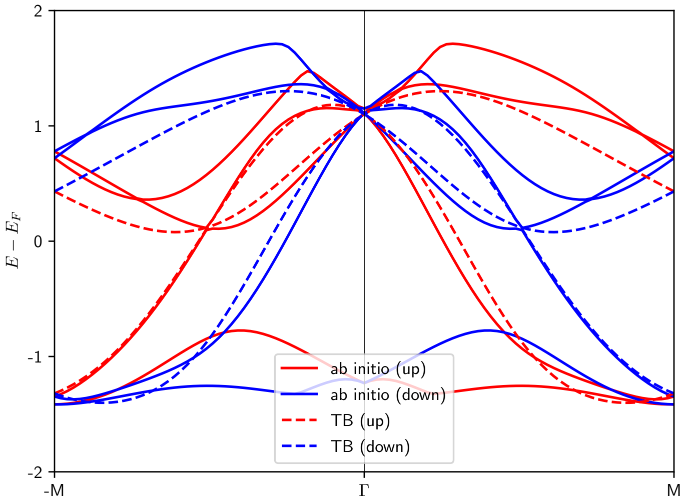

Fig. S3: Band structures along $\Gamma - M$ direction at $k_z = 0.28$. The solid (dashed) lines represent the band dispersion obtained from ab initio calculation (TB model). The red and blue colors represent spin-up and spin-down band sets, respectively.

approximatly by a set of hybridized orbitals, defined as follows,

$$\begin{aligned}
|\phi_1^1, Cr_1\rangle &= \frac{\sqrt{2}}{2}(-|d_{xz}, Cr_1\rangle - |d_{xy}, Cr_1\rangle) \\
|\phi_2^1, Cr_1\rangle &= \frac{\sqrt{2}}{2}(|d_{yz}, Cr_1\rangle + |d_{x^2-y^2}, Cr_1\rangle) \\
|\phi_1^2, Cr_1\rangle &= \frac{\sqrt{2}}{2}(-|d_{xz}, Cr_1\rangle + |d_{xy}, Cr_1\rangle) \\
|\phi_2^2, Cr_1\rangle &= \frac{\sqrt{2}}{2}(-|d_{yz}, Cr_1\rangle + |d_{x^2-y^2}, Cr_1\rangle) \\
|\phi_1^1, Cr_2\rangle &= \frac{\sqrt{2}}{2}(|d_{xz}, Cr_2\rangle - |d_{xy}, Cr_2\rangle) \\
|\phi_2^1, Cr_2\rangle &= \frac{\sqrt{2}}{2}(|d_{yz}, Cr_2\rangle - |d_{x^2-y^2}, Cr_2\rangle) \\
|\phi_1^2, Cr_2\rangle &= \frac{\sqrt{2}}{2}(|d_{xz}, Cr_2\rangle + |d_{xy}, Cr_2\rangle) \\
|\phi_2^2, Cr_2\rangle &= \frac{\sqrt{2}}{2}(|d_{yz}, Cr_2\rangle + |d_{x^2-y^2}, Cr_2\rangle)
\end{aligned} \tag{9}$$

where $|\phi_i^j, Cr_\alpha\rangle$ is the ith orbital basis of the jth hybridized E_g orbital on Cr_α. Using the orbital transformation in Eq. 9, we replot the orbital resolved band structures in Fig. S4. It shows that the bands crossing E_F are mainly contributed by the second E_g orbital on Cr_1 (consisting of $|\phi_1^2, Cr_1\rangle$ and $|\phi_2^2, Cr_1\rangle$) and the first E_g orbital on Cr_2 (consisting of $|\phi_1^1, Cr_2\rangle$ and $|\phi_2^1, Cr_2\rangle$). As obtained from the Wannier calculations (see Table 4 below), the direct 3NN hopping between them (i.e. $q_2 + q_3$ in Eq. 6) is about 21 meV, which is much smaller than the fitted value 173 meV. We also notice that the Wannier functions of p orbitals on Sb have a large spread and they hybridize significantly with the local d orbitals. Hence the large value of $q_2 + q_3$ should be dominated by a second-order hopping process mediated by Sb, or in other words, the p orbitals on Sb assist the

3NN hopping between d orbitals on Cr.

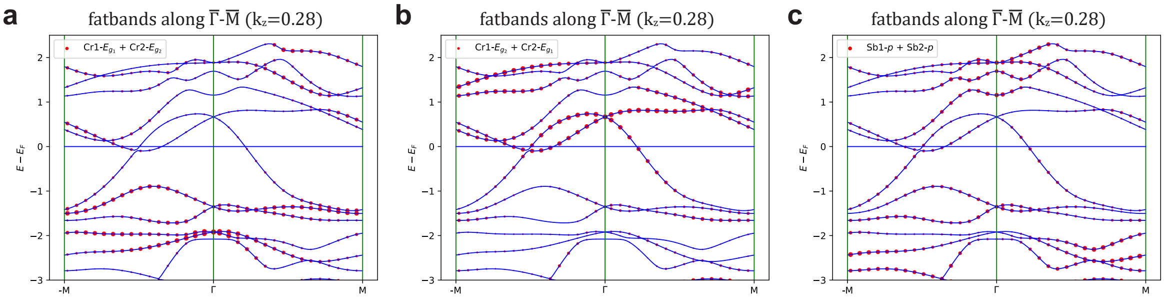

Fig. S4: The orbital-resolved band structures of the spin up component along $\Gamma - M$ direction at $k_z = 0.28$. The orbital weights of **a** $|\phi_1^2, Cr_2\rangle$,$|\phi_2^2, Cr_2\rangle$, $|\phi_1^1, Cr_1\rangle$ and $|\phi_2^1, Cr_1\rangle$ **b** $|\phi_1^2, Cr_1\rangle$,$|\phi_2^2, Cr_1\rangle$, $|\phi_1^1, Cr_2\rangle$ and $|\phi_2^1, Cr_2\rangle$, **c** $|p_z\rangle$,$|p_x\rangle$ and $|p_y\rangle$ on Sb, are plotted with red circles.

The assistant hopping between d orbitals from $\mathbf{r_i}$ to $\mathbf{r_j}$ and mediated by the p orbitals at $\mathbf{r_k}$ can be calculated in the second-order approximation,

$$\tilde{t}_{ij} \approx \frac{t_{ik} t_{kj}}{(\Delta_i - \Delta_k)(\Delta_k - \Delta_j)} \tag{10}$$

where t_{ik} is the direct hopping between d orbital at $\mathbf{r_i}$ and p orbital at $\mathbf{r_k}$, and $\Delta_{i,j,k}$ is the onsite energy of the corresponding orbital at $\mathbf{r_{i,j,k}}$. Using the second-order perturbation, we have calculated all the possible assistant hopping process between the d orbitals on Cr_1 at site $(0, 0, 0)$ and the d orbitals on Cr_2 at site $(1, 1, \frac{1}{2})$, and tabulated the ones contributing an assistant hopping larger than 10 meV in Table 3. It shows that the assistant hopping process mediated by the Sb at site $(\frac{1}{3}, \frac{2}{3}, \frac{1}{4})$, as schematically shown in Fig. S2b, is much more significant than the others. Indeed, it is the shortest path between the 3NN Cr atoms. By summing the direct hopping and all the assistant hopping obtained from the second-order perturbation, we derive the effective hopping values for q_1, $q_2 + q_3$, and q_4, as tabulated in Table 4. All of the values are approximately equal to the fitted parameters in Table 2.

Table 3: All the second-order processes that contribute an assistant hopping larger than 10 meV. The energy unit is eV.

second order process	t_{ik}	t_{kj}	Δ_i	Δ_k	Δ_j	$\tilde{t}_{ij}$
Cr[0,0,0] $\lvert\phi_1^2\rangle$ → Sb[-2/3,-1/3,1/4] px → Cr[1,1,1/2] $\lvert\phi_1^1\rangle$	-0.7343	-0.0443	8.1243	7.1126	9.9428	-0.0114
Cr[0,0,0] $\lvert\phi_1^2\rangle$ → Sb[-1/3,1/3,-1/4] pz → Cr[1,1,1/2] $\lvert\phi_1^1\rangle$	0.5125	0.0142	8.1243	7.8535	9.9428	-0.0128
Cr[0,0,0] $\lvert\phi_1^2\rangle$ → Sb[1/3,-1/3,1/4] px → Cr[1,1,1/2] $\lvert\phi_1^1\rangle$	-0.7343	0.0825	8.1243	7.1126	9.9428	0.0212
Cr[0,0,0] $\lvert\phi_1^2\rangle$ → Sb[4/3,2/3,1/4] pz → Cr[1,1,1/2] $\lvert\phi_1^1\rangle$	-0.0486	-0.2599	8.1243	7.8535	9.9428	-0.0223
Cr[0,0,0] $\lvert\phi_1^2\rangle$ → Sb[1/3,-1/3,1/4] pz → Cr[1,1,1/2] $\lvert\phi_2^1\rangle$	-0.5125	0.0245	8.1243	7.8535	9.9422	0.0222
Cr[0,0,0] $\lvert\phi_1^2\rangle$ → Sb[2/3,1/3,-1/4] pz → Cr[1,1,1/2] $\lvert\phi_2^1\rangle$	-0.5125	0.0263	8.1243	7.8535	9.9422	0.0238
Cr[0,0,0] $\lvert\phi_1^2\rangle$ → Sb[2/3,1/3,3/4] pz → Cr[1,1,1/2] $\lvert\phi_2^1\rangle$	0.0758	0.3003	8.1243	7.8535	9.9422	-0.0403
Cr[0,0,0] $\lvert\phi_1^2\rangle$ → Sb[4/3,2/3,1/4] pz → Cr[1,1,1/2] $\lvert\phi_2^1\rangle$	-0.0486	0.1502	8.1243	7.8535	9.9422	0.0129
Cr[0,0,0] $\lvert\phi_2^2\rangle$ → Sb[-1/3,-2/3,-1/4] pz → Cr[1,1,1/2] $\lvert\phi_1^1\rangle$	0.5915	0.0184	8.1231	7.8535	9.9428	-0.0193
Cr[0,0,0] $\lvert\phi_2^2\rangle$ → Sb[1/3,-1/3,1/4] px → Cr[1,1,1/2] $\lvert\phi_1^1\rangle$	0.4282	0.0825	8.1231	7.1126	9.9428	-0.0124
Cr[0,0,0] $\lvert\phi_2^2\rangle$ → Sb[1/3,2/3,1/4] pz → Cr[1,1,1/2] $\lvert\phi_1^1\rangle$	-0.5915	0.2599	8.1231	7.8535	9.9428	0.2728
Cr[0,0,0] $\lvert\phi_2^2\rangle$ → Sb[1/3,2/3,1/4] py → Cr[1,1,1/2] $\lvert\phi_1^1\rangle$	-0.9812	-0.3392	8.1231	7.1119	9.9428	-0.1162
Cr[0,0,0] $\lvert\phi_2^2\rangle$ → Sb[2/3,4/3,3/4] pz → Cr[1,1,1/2] $\lvert\phi_1^1\rangle$	-0.0369	0.2599	8.1231	7.8535	9.9428	0.017
Cr[0,0,0] $\lvert\phi_2^2\rangle$ → Sb[4/3,2/3,1/4] pz → Cr[1,1,1/2] $\lvert\phi_1^1\rangle$	-0.0282	-0.2599	8.1231	7.8535	9.9428	-0.013
Cr[0,0,0] $\lvert\phi_2^2\rangle$ → Sb[1/3,-1/3,1/4] pz → Cr[1,1,1/2] $\lvert\phi_2^1\rangle$	0.2957	0.0245	8.1231	7.8535	9.9422	-0.0129
Cr[0,0,0] $\lvert\phi_2^2\rangle$ → Sb[2/3,1/3,-1/4] pz → Cr[1,1,1/2] $\lvert\phi_2^1\rangle$	-0.2957	0.0263	8.1231	7.8535	9.9422	0.0138
Cr[0,0,0] $\lvert\phi_2^2\rangle$ → Sb[1/3,2/3,1/4] pz → Cr[1,1,1/2] $\lvert\phi_2^1\rangle$	-0.5915	0.1502	8.1231	7.8535	9.9422	0.1577
Cr[0,0,0] $\lvert\phi_2^2\rangle$ → Sb[1/3,2/3,1/4] py → Cr[1,1,1/2] $\lvert\phi_2^1\rangle$	-0.9812	0.2919	8.1231	7.1119	9.9422	0.1001
Cr[0,0,0] $\lvert\phi_2^2\rangle$ → Sb[2/3,1/3,3/4] pz → Cr[1,1,1/2] $\lvert\phi_2^1\rangle$	0.0435	0.3003	8.1231	7.8535	9.9422	-0.0232

Table 4: The direct, assitant, and effective (total) hopping values of parameters $q_1, q_2 + q_3, q_4$. The energy unit is eV.

	q_1	$q_2 + q_3$	q_4
t_{direct}	0.005	0.0214	-0.066
$\tilde{t}$	-0.0234	0.1516	0.2602
$t_{eff} = t_{direct} + \tilde{t}$	-0.0184	0.1730	0.1942

3 Matrix representation of the crystalline symmetries

In this section, we provide the matrix representations of all the symmetries $\hat{R}$ that leave the Hamiltonian $\hat{H}_{\uparrow}$ in Eq. (3) invariant,

$$\hat{H}_{\uparrow} = \hat{R}^{-1} \hat{H}_{\uparrow} \hat{R} \tag{11}$$

We still use the atomic basis as defined in Eq. (1).

Identity operation $\hat{E}$:

$$\hat{E} = \begin{pmatrix} 1 & 0 & 0 & 0 \\ 0 & 1 & 0 & 0 \\ 0 & 0 & 1 & 0 \\ 0 & 0 & 0 & 1 \end{pmatrix}$$

The two three-fold rotation symmetry along the z-axis $\hat{C}_{3z}^{i}$:

$$\hat{C}_{3z}^{1} = \begin{pmatrix} -\frac{1}{2} & -\frac{\sqrt{3}}{2} & 0 & 0 \\ \frac{\sqrt{3}}{2} & -\frac{1}{2} & 0 & 0 \\ 0 & 0 & -\frac{1}{2} & -\frac{\sqrt{3}}{2} \\ 0 & 0 & \frac{\sqrt{3}}{2} & -\frac{1}{2} \end{pmatrix}$$

$$\hat{C}_{3z}^{2} = \begin{pmatrix} -\frac{1}{2} & \frac{\sqrt{3}}{2} & 0 & 0 \\ -\frac{\sqrt{3}}{2} & -\frac{1}{2} & 0 & 0 \\ 0 & 0 & -\frac{1}{2} & \frac{\sqrt{3}}{2} \\ 0 & 0 & -\frac{\sqrt{3}}{2} & -\frac{1}{2} \end{pmatrix}$$

The two-fold rotation symmetry along the x-axis $\hat{C}_{2x}$:

$$\hat{C}_{2x} = \begin{pmatrix} -1 & 0 & 0 & 0 \\ 0 & 1 & 0 & 0 \\ 0 & 0 & -1 & 0 \\ 0 & 0 & 0 & 1 \end{pmatrix}$$

Inversion symmetry $\hat{P}$:

$$\hat{P} = \begin{pmatrix} 1 & 0 & 0 & 0 \\ 0 & 1 & 0 & 0 \\ 0 & 0 & 1 & 0 \\ 0 & 0 & 0 & 1 \end{pmatrix}$$

All the generators of unitary-operation $\hat{R}$ are listed above. The anti-unitary operations satisfied by $\hat{H}_{\uparrow}$ are $\hat{R}\hat{T}$, where $\hat{T}$ is the time-reversal operation:

$$\hat{T} = \begin{pmatrix} 1 & 0 & 0 & 0 \\ 0 & 1 & 0 & 0 \\ 0 & 0 & 1 & 0 \\ 0 & 0 & 0 & 1 \end{pmatrix} \hat{K}$$

where $\hat{K}$ is the complex conjugate operation: $\hat{K}c = c^{*}\hat{K}$, c is a complex number.

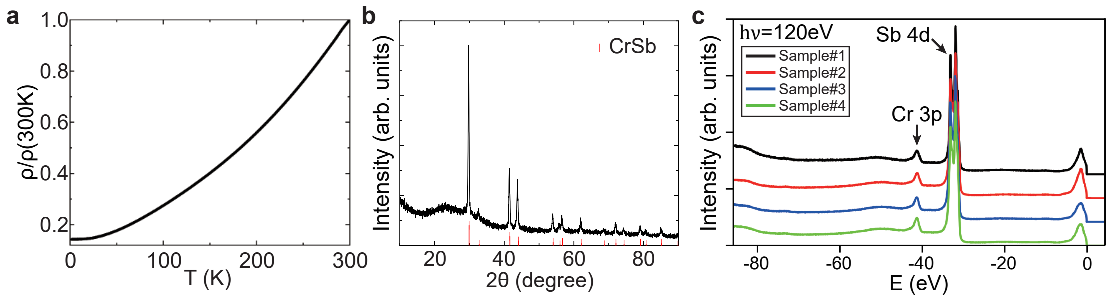

Fig. S5: **Additional characterizations of CrSb.** **a** Normalized resistivity ($\rho/\rho(300\text{ K})$) as a function of temperature for a typical CrSb single crystal. **b** Powder XRD pattern from a polycrystal CrSb sample (black curve). The expected peaks for CrSb are shown at the bottom. **c** Core level scans taken with 120 eV photons from four different samples, showing the Cr $3p$ and Sb $4d$ peaks without any trace of impurities. The strong Sb $4d$ peaks imply possibly Sb-terminated surface.

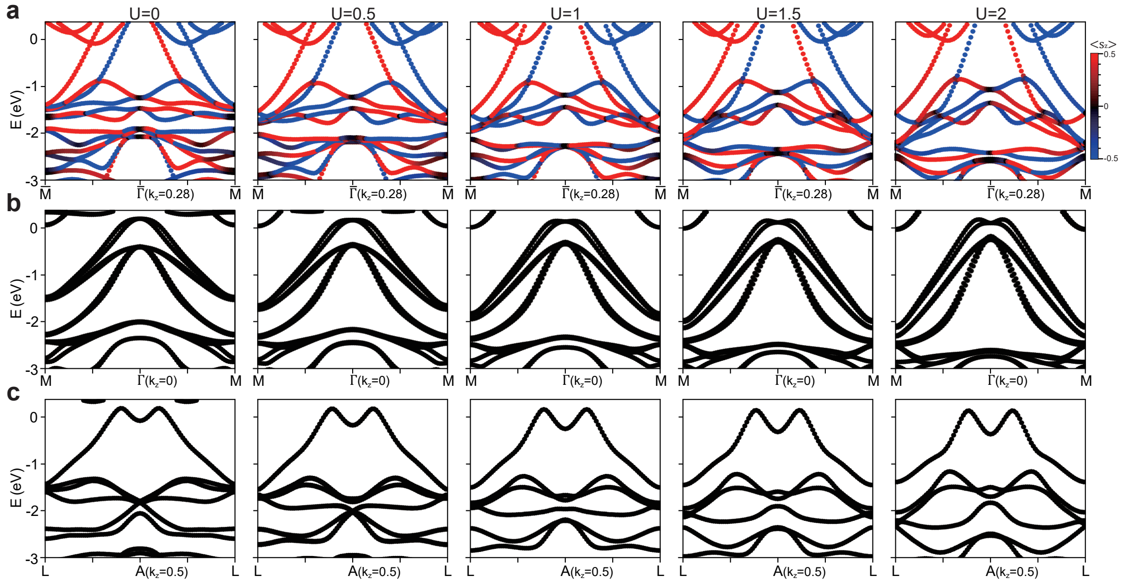

Fig. S6: Calculated band structure along $\bar{M}-\bar{\Gamma}-\bar{M}$ at $k_z = 0.28c^*$ (**a**), 0 (**b**) and $0.5c^*$ (**c**). From left to right: calculation results with the Coulomb repulsion U set to 0, 0.5, 1, 1.5 and 2 eV. Since the $U = 0$ eV calculation yields best agreement with ARPES data, we choose $U = 0$ eV for all the calculations in the paper.

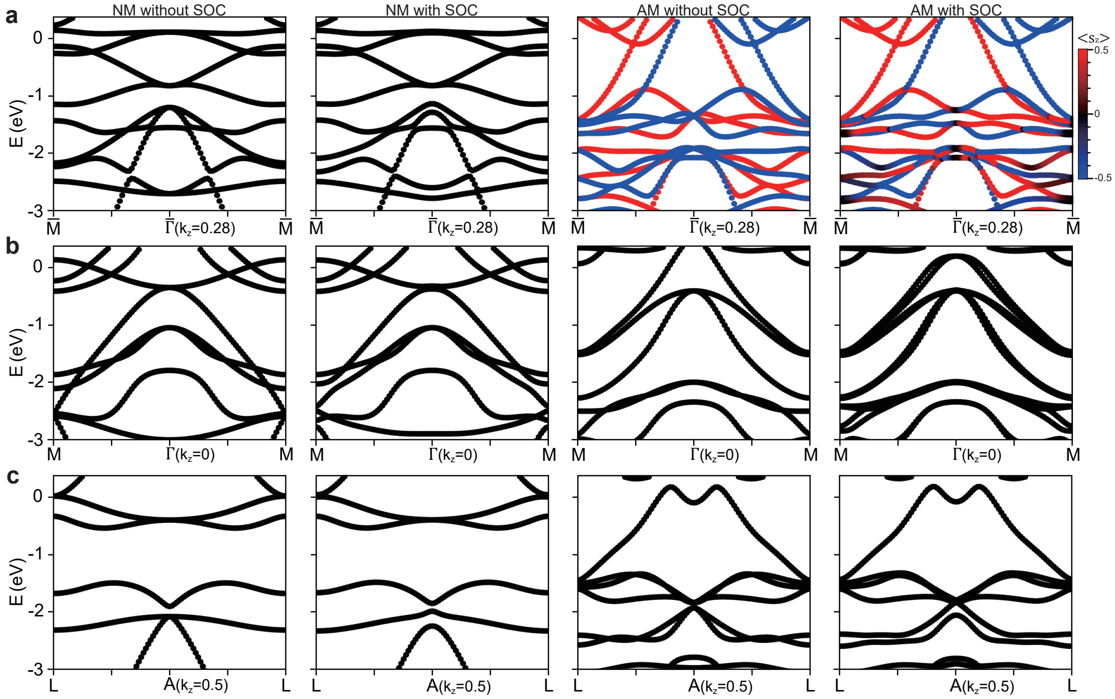

Fig. S7: Calculated band structure for CrSb at $k_z = 0.28c^*$ (**a**), 0 (**b**) and $0.5c^*$ (**c**). From left to right: calculation ignoring both altermagnetism and SOC [nonmagnetic (NM) without SOC], ignoring altermagnetism but considering SOC (NM with SOC), considering altermagnetism but ignoring SOC [altermagnetism (AM) without SOC], considering both altermagnetism and SOC [AM with SOC]. The colors of the curves indicate spin polarization (color bar on the right).

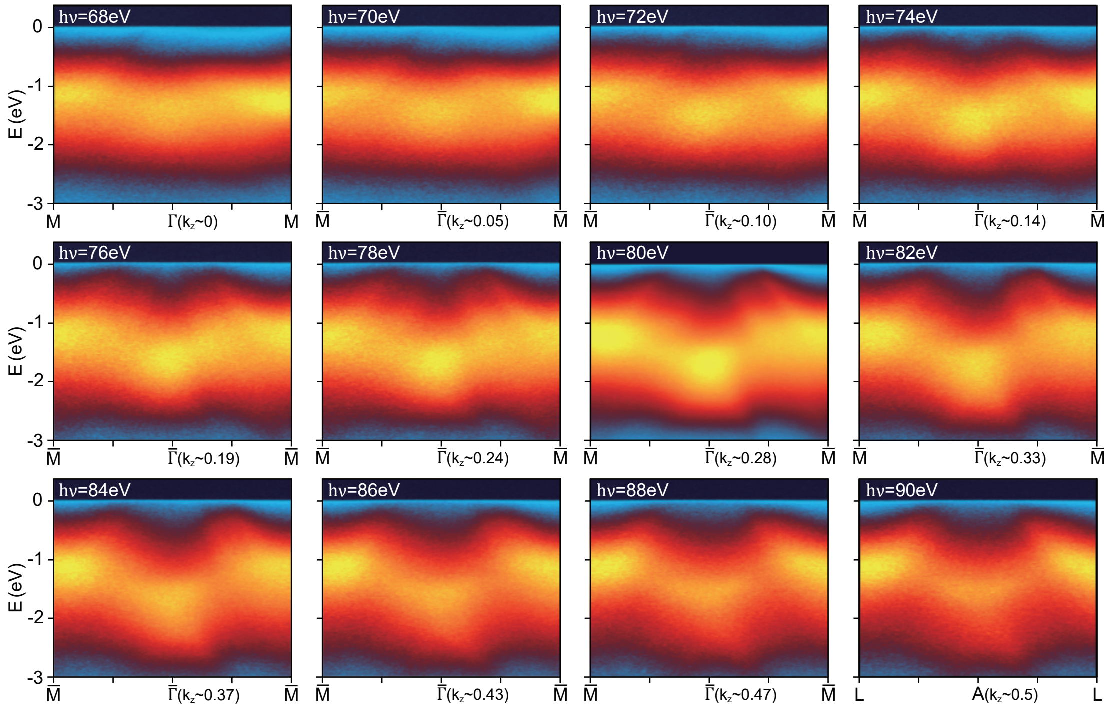

Fig. S8: The ARPES spectra along the $\bar{M} - \bar{\Gamma} - \bar{M}$ direction taken with different photon energies. The corresponding k_z values (in units of c^*) are also indicated.

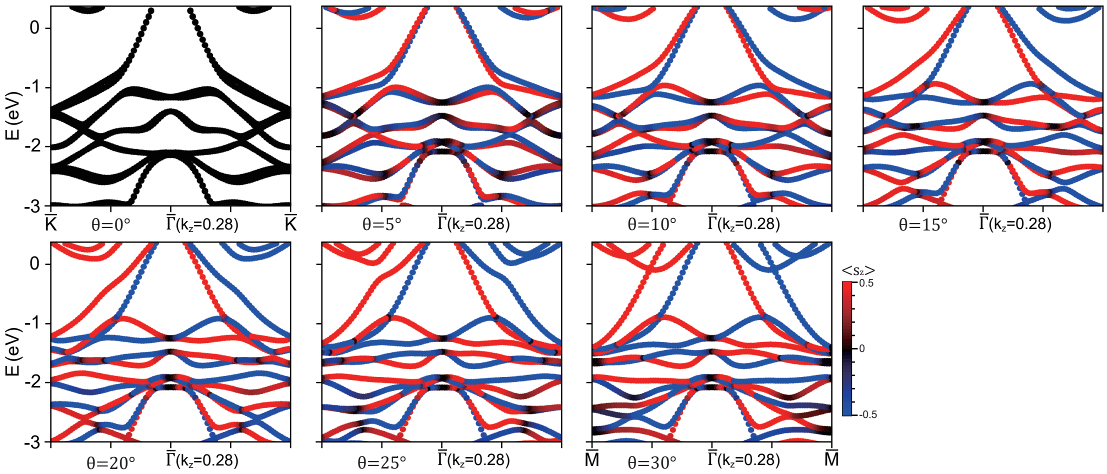

Fig. S9: Calculated in-plane band dispersion at $k_z \sim 0.28c^*$ for different θ angles (defined in Fig. **3a**). The altermagnetic splitting shows a monotonic increase from $\bar{K}-\bar{\Gamma}-\bar{K}$ ($\theta = 0°$) to $\bar{M}-\bar{\Gamma}-\bar{M}$ ($\theta = 30°$).

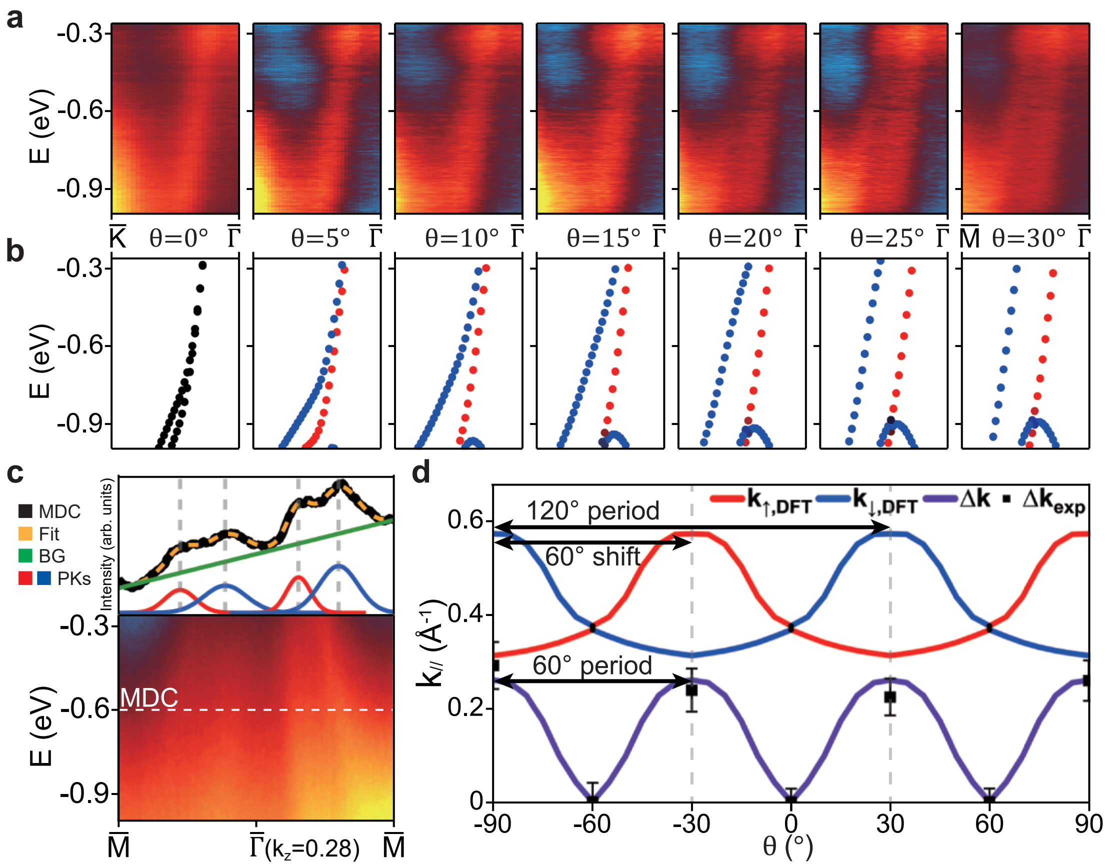

Fig. S10: **Additional ARPES data and analysis illustrating the in-plane altermagnetic splitting. a** Energy-momentum cuts for different θ angles with a small step of 5°, extracted from Fig. 3**a**. **b** Band structure from DFT for comparison with (**a**). **c** Illustration of peak fitting from the momentum distribution curve (MDC). A Gaussian shape is assumed for all the peaks and a smooth background is included. The raw data, fitting result (Fit), background (BG) and Gaussian peaks (PKs) are shown respectively. **d** The in-plane momentum of up-spin ($k_{\uparrow,DFT}$, red curve) and down-spin ($k_{\downarrow,DFT}$, blue curve) bands at E = -0.6 eV as a function of θ from DFT. The purple curve is the magnitude of spin splitting ($\Delta k = | k_{\uparrow,DFT} - k_{\downarrow,DFT} |$). The black boxes with error bars are experimental results at high-symmetry points.

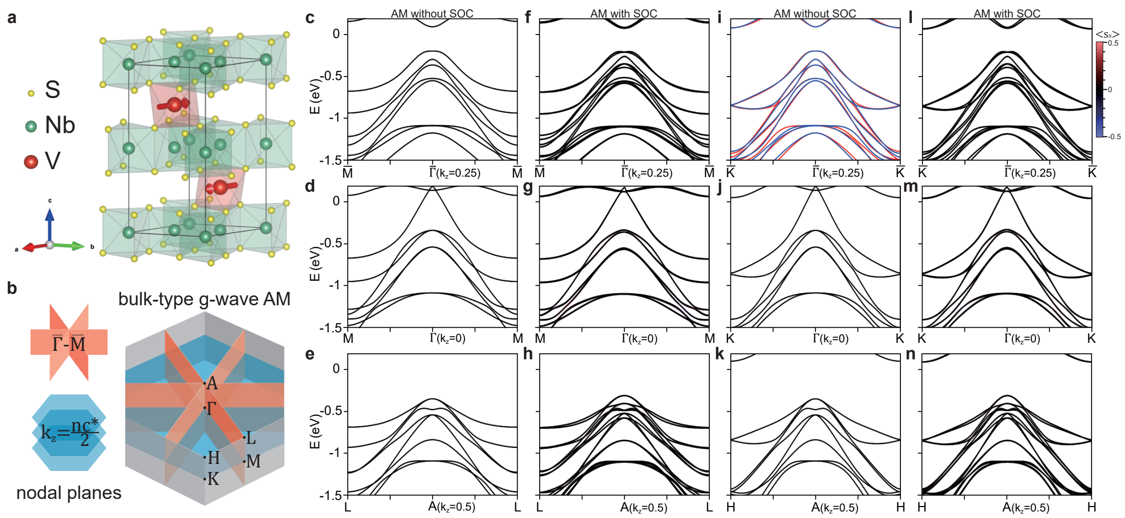

Fig. S11: **Bulk-type g-wave altermagnet VNb_3S_6 with much smaller splitting than CrSb.** **a** Spin and lattice structure of VNb_3S_6. **b** The BZ with high symmetry points and nodal planes labelled. Three equivalent nodal planes parallel to k_z are along $\bar{M} - \bar{\Gamma} - \bar{M}$. **c-n** DFT calculations along $\bar{\Gamma} - \bar{M}$ (**c-h**) and $\bar{\Gamma} - \bar{K}$ (**i-n**) at $k_z = 0.25$ (top row), 0 (middle row), 0.5 (bottom row) c^*, respectively. Calculations with SOC (**f,g,h,l,m,n**) and without SOC (**c,d,e,i,j,k**) are both considered. SOC has only minor effect on the band dispersion, despite reducing the spin polarization. The magnitude of altermagnetic splitting is about a few tens of meV, much smaller than CrSb.

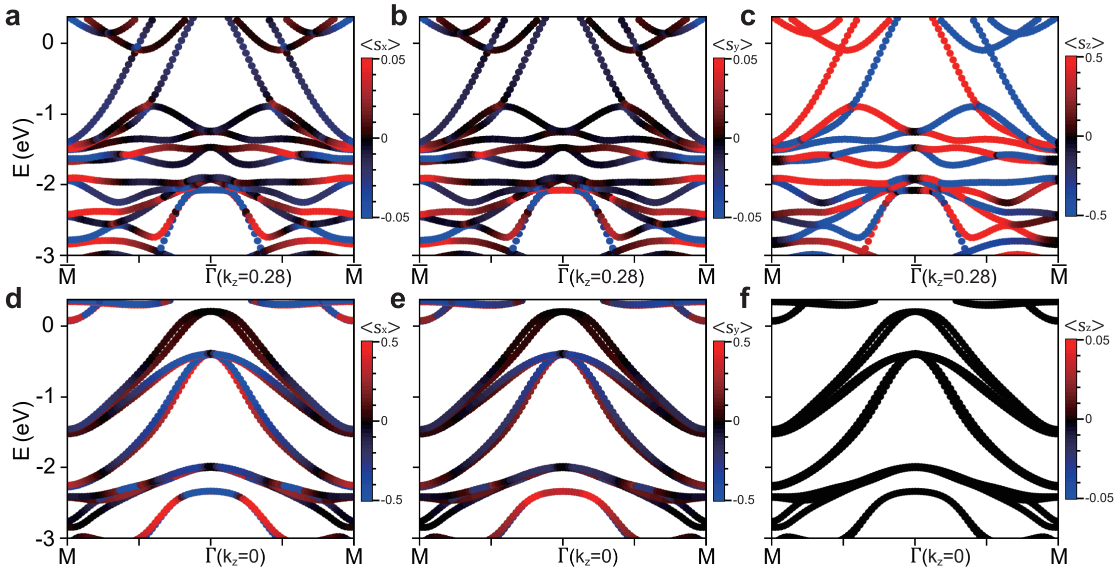

Fig. S12: **Spin polarizations of DFT bands along x, y and z directions considering both altermagnetism and SOC. a-c** Spin polarizations along x (**a**, $< S_x >$), y (**b**, $< S_y >$) and z (**c**, $< S_z >$) directions, for bands along $\bar{\Gamma} - \bar{M}$ at $k_z = 0.28c^*$. Please note the much larger color scales (shown on the right) for (**c**) compared to (**a-b**). For bands away from E_F, i.e., $E \sim$ -2.5 eV where appreciable contributions from Sb p orbitals are present, the inclusion of SOC makes $< S_z >$ deviate from the quantized value of $\pm$ 1/2, leading to small $< S_x >$ and $< S_y >$ components shown in (**a-b**). However, for bands near E_F, $< S_z >$ is still close to $\pm$ 1/2, indicating weak SOC effects. **d-f** Spin polarizations along x (**d**, $< S_x >$), y (**e**, $< S_y >$) and z (**f**, $< S_z >$) directions, for bands along $\bar{\Gamma} - \bar{M}$ at $k_z = 0$. Without SOC, this k_z plane is a nodal plane without altermagnetic splitting. The inclusion of SOC induces a very tiny splitting (see also Fig. S7). While the spin polarization along the z direction remains zero (**f**), protected by $[C_2^s \| M_z]$, there is a very small in-plane spin polarization due to SOC (**d,e**). This is similar to the weak altermagnetic splitting discussed in [13].

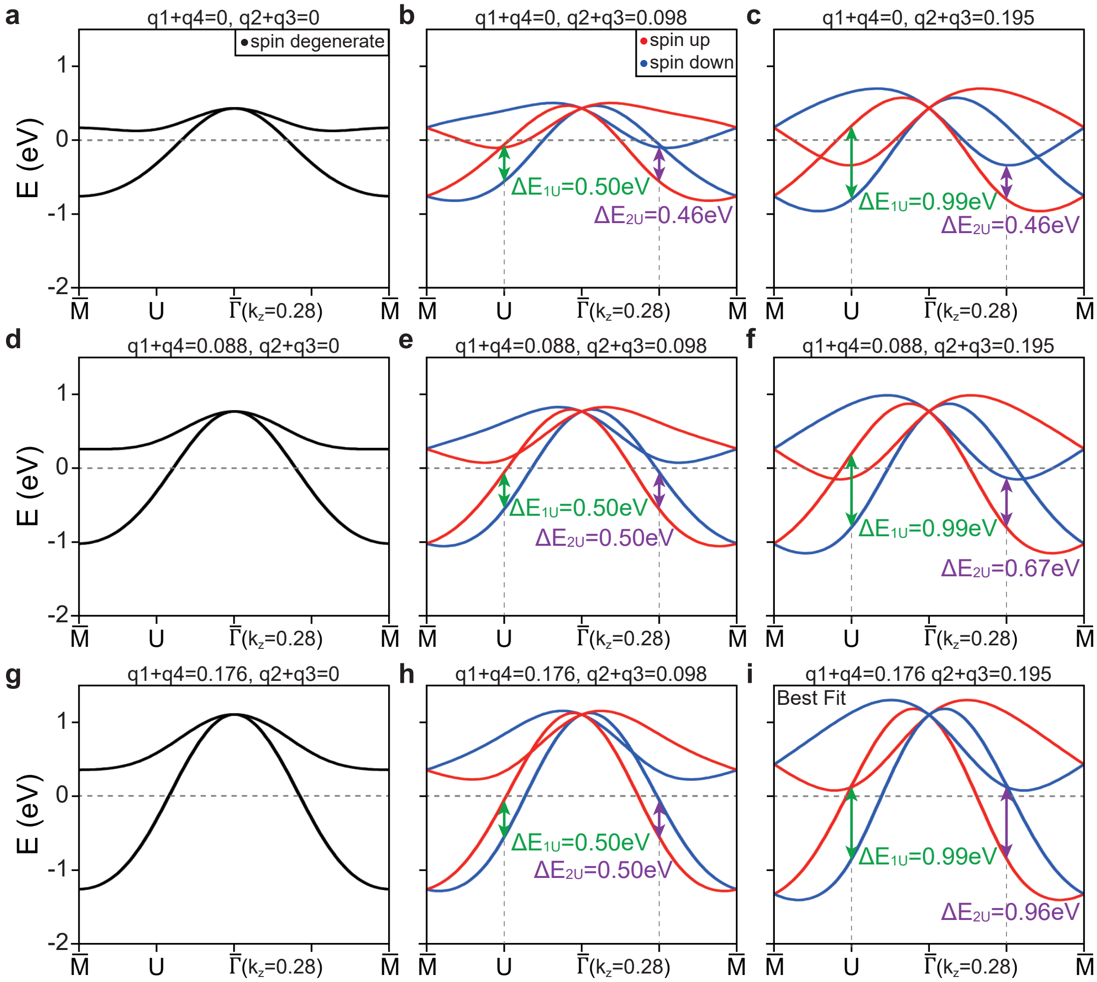

Fig. S13: **Dependence of band dispersion on different 3NN hopping parameters.** Band dispersions along the $\bar{\Gamma} - \bar{M}$ direction at $k_z = 0.28c^*$ are obtained from the eight-band TB model using different $q_1 + q_4$ and $q_2 + q_3$ values, as indicated at the top of each figure. The red, blue and black lines represent spin-up, spin-down and spin-degenerate bands, respectively.
